# Probiotics, prebiotics infant formula use in preterm or low birth weight infants: a systematic review

**DOI:** 10.1186/1475-2891-11-58

**Published:** 2012-08-28

**Authors:** Mary N Mugambi, Alfred Musekiwa, Martani Lombard, Taryn Young, Reneé Blaauw

**Affiliations:** 1 Division of Human Nutrition, Faculty of Medicine and Health Sciences, Stellenbosch University, P.O Box 19063, Tygerberg 7505, South Africa; 2 Wits Reproductive Health & HIV Institute (WRHI), Faculty of Health Sciences, University of the Witwatersrand, Johannesburg, South Africa; 3 Centre for Evidence-Based Health Care, Faculty of Medicine and Health Sciences, Stellenbosch University, Tygerberg, South Africa

**Keywords:** Probiotic, Prebiotic, Preterm infant, Low birth weight infant

## Abstract

**Background:**

Previous reviews (2005 to 2009) on preterm infants given probiotics or prebiotics with breast milk or mixed feeds focused on prevention of Necrotizing Enterocolitis, sepsis and diarrhea. This review assessed if probiotics, prebiotics led to improved growth and clinical outcomes in formula fed preterm infants.

**Methods:**

Cochrane methodology was followed using randomized controlled trials (RCTs) which compared preterm formula containing probiotic(s) or prebiotic(s) to conventional preterm formula in preterm infants. The mean difference (MD) and corresponding 95% confidence intervals (CI) were reported for continuous outcomes, risk ratio (RR) and corresponding 95% CI for dichotomous outcomes. Heterogeneity was assessed by visual inspection of forest plots and a chi^2^ test. An I^2^ test assessed inconsistencies across studies. I^2^> 50% represented substantial heterogeneity.

**Results:**

Four probiotics studies (N=212), 4 prebiotics studies (N=126) were included. **Probiotics:** There were no significant differences in weight gain (MD 1.96, 95% CI: -2.64 to 6.56, 2 studies, n=34) or in maximal enteral feed (MD 35.20, 95% CI: -7.61 to 78.02, 2 studies, n=34), number of stools per day increased significantly in probiotic group (MD 1.60, 95% CI: 1.20 to 2.00, 1 study, n=20). **Prebiotics:** Galacto-oligosaccharide / Fructo-oligosaccharide (GOS/FOS) yielded no significant difference in weight gain (MD 0.04, 95% CI: -2.65 to 2.73, 2 studies, n=50), GOS/FOS yielded no significant differences in length gain (MD 0.01, 95% CI: -0.03 to 0.04, 2 studies, n=50). There were no significant differences in head growth (MD −0.01, 95% CI: -0.02 to 0.00, 2 studies, n=76) or age at full enteral feed (MD −0.79, 95% CI: -2.20 to 0.61, 2 studies, n=86). Stool frequency increased significantly in prebiotic group (MD 0.80, 95% CI: 0.48 to 1.1, 2 studies, n=86). GOS/FOS and FOS yielded higher bifidobacteria counts in prebiotics group (MD 2.10, 95% CI: 0.96 to 3.24, n=27) and (MD 0.48, 95% CI: 0.28 to 0.68, n=56).

**Conclusions:**

There is not enough evidence to state that supplementation with probiotics or prebiotics results in improved growth and clinical outcomes in exclusively formula fed preterm infants.

## Background

Growth is a major challenge for premature and low birth weight infants (born < 37 weeks gestation or with a birth weight of < 2500 g). They have several factors that put them at risk for nutritional deficiencies resulting in poor growth. Decreased nutrient stores result in low body stores of glycogen, fat, protein, fat soluble vitamins, calcium, phosphorus, magnesium and trace minerals. Preterm infants require increased energy and nutrients for rapid growth and may need a 10 fold increase in weight gain in order to achieve optimum catch up growth [1,2]. To achieve optimum growth for the preterm infant, the goals are to continue the process of intra-uterine growth in an extra-uterine environment until 40 weeks post conception, foster catch-up growth and nutrient accumulation in the post discharge period [3-6]. A weight gain of 15 to 20 g/ kg/day, length of 0.75 to 1.0 cm/week and head circumference 0.75 cm/week is required. This is difficult to achieve and requires between 130 – 135 kcal / kg /day to maintain this growth rate [3]. Furthermore, infants lose weight after birth (up to 6% to 8% for extreme low birth weight infants) and they often do not regain the weight for up to 1 to 2 weeks [5]. Daily growth monitoring (weight gain, linear and head circumference) then becomes vital.

Preterm infants have immature physiological systems due to an underdeveloped gastrointestinal barrier function as reflected by increased intestinal permeability. As a result, potentially pathogenic bacteria translocate from the intestinal lumen and cause systemic infections [7]. Reducing intestinal permeability is associated with gut maturation which promotes growth and avoids severe infections [4]. In addition, digestive and absorptive capabilities are decreased due to low concentration of lactase, pancreatic lipase and bile salts. Gastrointestinal motility and stomach capacity are decreased which limits feeding volume and gastric emptying. A coordinated suck and swallow is not developed until 32 to 34 weeks gestation. Introduction of enteral feeding maybe delayed due to increased risk of aspiration [1,2,8,9]. Preterm infants in neonatal intensive care units (NICUs) develop a different intestinal microbiota compared to healthy breast fed infants. This is due to decreased exposure to the maternal microbiota, increased exposure to organisms that colonize NICUs, multiple courses of antibiotics and delays in feeding [8,9].

Humans have consumed probiotics in the form of fermented food, dairy products and more recently infant and toddler formula. Probiotics are defined as “live microorganisms” which when administered in adequate amounts confer a health benefit to the host [10]. The main probiotic organisms used worldwide belong to the genera Lactobacillus and Bifidobacteria and are found in the gastrointestinal micro flora [10,11]. Prebiotics are found in fruit and vegetable components, they are non- digestible food ingredients that benefit the host by selectively stimulating the growth and/or activity of one or a limited number of bacteria in the colon and improving the host’s health [12,13]. The most widely studied prebiotics are inulin, fructo-oligosaccharide (FOS) and galacto-oligosaccharide (GOS) which are plant storage carbohydrates in vegetables, cereals and fruit. FOS and inulin are added to different foods as fat and sugar replacements to improve texture or for their functional benefits [12,14-16]. Probiotics and prebiotics are added to infant formula to promote an intestinal microbiota resembling that of breastfed infants which have a greater concentration of bifidobacteria and less pathogenic bacteria than formula fed infants [10,17].

There are a number of ways in which probiotics improve health. Health benefits conferred by probiotic bacteria are strain specific and not species or genus specific [10]. Probiotic bacteria improve health by affecting the immune system in different ways. They increase cytokine production such as Interleukin-6 (IL-6), Interferon- gamma (IFN-γ), Tissue Necrosis Factor – alpha (TNF-α), Interleukin-1beta (IL-1β) and Interleukin-10 (IL-10) [18]. Some strains increase phagocytic activity of peripheral blood leukocytes (monocytes, polymorphonuclear cells). Other strains strengthen the mucosal barrier function by promoting the production of mucosal antibodies and reducing the trans mucosal transfer of antigens. This reduces the intestinal permeability which in turn promotes growth [19-22]. Probiotics bacteria also enhance production of low molecular weight antibacterial substances produced by epithelial cells and production of short chain fatty acids, the main energy source for colonocytes. This maintains the integrity of colon mucosa [19,23-26].

Prebiotics are resistant to digestive enzymes and pH extremes found in the human gastrointestinal tract. They transit through the upper gastrointestinal tract and reach the colon intact where they are selectively fermented by indigenous bacteria, especially bifidobacteria and lactobacilli [12,15,26,27]. Beneficial bacteria (including bifidobacteria and lactobacilli) possess enzymes needed to metabolize prebiotics, while other bacteria (such as E coli, clostridia and salmonella) do not [15,27]. Consumption of prebiotics by preterm formula fed infants results in an increase of beneficial microorganisms in the colon, decreasing harmful bacteria to the levels found in breastfed infants. This improves the gastrointestinal mucosal barrier (decreasing intestinal permeability) which prevents infections and eventually results in improved growth [27,28]. In general the aim of adding probiotics and prebiotics to preterm infant formula is to improve growth, development and decrease infections by promoting an intestinal microbiota resembling that of breastfed infants [9,29,30].

The effects of probiotics on the intestinal microbiota of premature infants have been varied due to differences on gestational age and products administered. Effects of probiotics on weight gain have also been varied. Administration of *Bifidobacteria breve* led to improved weight gain while *Saccharomyces bourladii* did not [9]. With premature infants optimal strains and dose regimens are yet to be examined closely [8]. The effects of prebiotics on the growth of premature infants are not clear. If prebiotic supplementation reduces the risk of Necrotizing Enterocolitis (NEC) or improves feed tolerance in very low birth weight infants is yet to be established [8,9]. Recent systematic reviews (published from 2005 to 2009) on the use of probiotics or prebiotics in preterm infants have focused on prevention of NEC and / or sepsis, impact on diarrhea [31-34]. These reviews focused on studies that used breast milk and mixed feeds (formula combined with breast milk). This review included infants given only infant formula and focused on growth with clinical outcomes that were not adequately addressed by previous reviews.

The Human Research Ethics Committee at the University of Stellenbosch, South Africa reviewed the review protocol (unpublished), ruled that all data to be collected for this review was from the public domain and was therefore exempt from ethical approval.

## Objective

To assess if addition of probiotics or prebiotics to preterm infant formula led to improved growth and clinical outcomes in preterm or low birth weight infants.

## Methods

### Eligibility criteria

All randomized controlled trials (RCTs), irrespective of language, which compared the use of preterm infant formula containing probiotic(s) or prebiotic(s) to conventional preterm infant formula without or with placebo amongst preterm infants born <37 weeks gestation, low birth weight infants with <2.5 kg at birth and hospitalized, receiving formula feeds and / or parenteral feed were considered. Studies published as abstracts were included if sufficient information could be obtained to assess study quality and obtain relevant study findings.

### Outcome measurements

Primary outcomes included: Short term growth parameters (assessed for entire study duration approximately 4 weeks): weight gain (grams/day or grams/week), linear growth (centimeters/week), head growth (cm/week). Secondary outcomes included: Complications: Incidence of NEC (defined as suspected or confirmed positive Bell stage II or more), Sepsis (defined as signs or symptoms of infection and positive blood culture), Other infections (example bacteraemia defined as blood cultured positive for bacteria), Mortality / death. Adverse events during entire study duration: Number of days on parenteral, number of days to full enteral nutrition, maximal enteral feed (millilitres/day, millilitres/kilogram/day, millilitres /kilogram). Feed intolerance: Incidence of vomiting, gastric aspirates, abdominal distension. Stool characteristics: Stooling frequency and stool consistency as firm, loose or watery. Changes in intestinal permeability as measured by ratio of Lactulose / mannitol in urine or other sugar absorption tests (such as lactulose / L – rhamnose ratio, D- xylose, 3-*O2*- methyl-D- glucose tests). Gastrointestinal (GI) micro flora: number of colony forming units (cfu) of bifidobacteria, lactobacillus and pathogens post intervention).

### Search method for identification of studies

A literature search in all languages was conducted on electronic databases which included The Cochrane Central Register for Controlled Trials 2009, Scopus (1990 to 19/01/2010), EBSCO host (1960 to 15/11/2009), OVID (1950 to 01/12/2009), SPORT Discus (1960 to 19/01/2010), Web of Science (1970 to 19/01/2010), Science Direct (1950 to 30/11/2009), EMBASE (1980 to 01/12/2009), CINAHL (1981 to 19/01/2010), PUBMED / MEDLINE (1966 to 10/04/2010), Latin American Caribbean Health Sciences literature (LILACS), (1965 to 19/01/2010), NLM Gateway (1950–1966). RCTs published in non-English language journals were translated by independent translators who were familiar with the subject matter. The search strategy used to search PUBMED is shown on Table 1. This search strategy was modified to search other electronic databases.

**Table 1:** Search strategy used in PUBMED

1)	Search (probiotic* OR prebiotic*) AND (infant formula* OR infant feeding OR formula OR formula milk) AND (preterm or premature or low birth weight babies) AND (randomized controlled trial* OR controlled clinical trial* OR random allocation*) Limits: Human
2)	Search (probiotic* infant formula* OR prebiotic* infant formula* OR prebiotic* OR probiotic*) AND (infant formula* OR infant feeding) AND (premature OR preterm) AND (randomized controlled trial* OR controlled clinical trial OR random allocation* OR double blind method OR single-blind method OR clinical trial OR placebo* OR random* OR research design OR comparative study OR follow-up studies OR prospectiv* OR volunteer* OR control* (singl* OR doubl* OR trebl* OR tripl*) NEAR (blind* OR mask*) Limits: Human

We conducted a hand search on abstracts of major conference proceedings such as the Pediatric Academic Society meetings (www.pas-meetings.org, www.abstracts2view.com), cross checked references cited in RCTs and in recent reviews (published from 2005 to 2009) for additional studies not identified by electronic searches and specialty journals which were not included in any database such as Pediatrika, Chinese Journal of Microecology and International Journal of Probiotics and Prebiotics.

To identify on-going and unpublished trials, we contacted experts in the field, manufacturers of infant formula containing probiotics and prebiotics, we searched web sites of companies that have conducted or were conducting RCTs on probiotics and prebiotics e.g. Pfizer (www.pfizerpro.com/clinicaltrials), Chris Hansen Laboratory (www.chr-hansen.com/research_development/documentation.html). We also searched prospective trial registries such as World Health Organisation (WHO) International Clinical Trials Registry Platform Search Portal (www.who.int/trialsearch), Clinical Trials.gov register (www.clinicaltrials.gov), Current Controlled Trials *metaR*egister of Controlled Trials [*mRCT*] (www.controlled-trials.com/mrct) and www.clinicaltrialresults.org.

### Selection of studies

**Figure 1:**
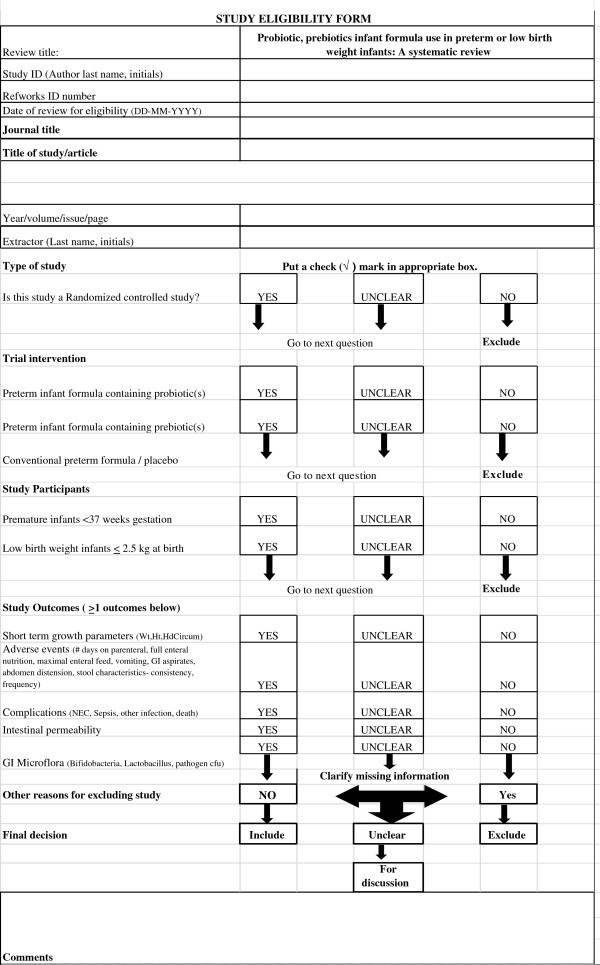
Study eligibility form.

Two reviewers (MM, ML) independently reviewed all abstracts, citations and identified potentially eligible studies. The full reports of eligible studies were retrieved by one reviewer (MM) and the pre-specified selection criteria applied independently by two reviewers (MM, ML) using a study eligibility form. (Figure 1) If more than one publication of a study existed, all reports of the study were grouped together under one study name. Any disagreements between the reviewers were resolved through discussion. If disagreements could not be resolved a third party was consulted. Trial authors were contacted if eligibility was unclear.

### Assessment of quality of evidence

Two reviewers (MM, ML) independently assessed the risk of bias of included studies as described in the Cochrane Handbook for Systematic Reviews for Interventions according to the following 6 components. 1) sequence generation; 2) allocation concealment; 3) blinding; 4) incomplete outcome data; 5) selective outcome reporting; and 6) other sources of bias [35]. Where necessary, trial authors were contacted for clarification on the methodology of their studies. Any disagreements regarding risk of bias were resolved through discussion between MM, ML and RB.

### Data extraction and management

Two reviewers (MM, ML) independently extracted data using a pre tested data extraction form. The reviewers (MM, ML) cross checked data and resolved any differences through discussion. One reviewer (MM) entered the data in Review Manager (RevMan 5) and the other reviewer (ML) validated the data. Trial authors were contacted for missing data or for clarification.

### Data synthesis and management

Results for probiotic and prebiotic studies were analysed separately. For continuous outcomes the mean difference (MD) and corresponding 95% confidence intervals (CI) were calculated. For dichotomous outcomes, the risk ratio (RR) and corresponding 95% CI were calculated. Trial authors were contacted if there was missing data in their reports. Available case analysis was used where there was missing data. The potential impact of the missing data on the results of the review is addressed in the discussion section. Heterogeneity of the trials used in the review was assessed by visually inspecting the forest plots to detect overlapping confidence intervals and by performing a chi^2^ test. A p<0.1 was considered statistically significant. An I-square test (I^2^) was used to test for inconsistencies across studies. If the I^2^ exceeded 50% and visual inspection of the forest plot supported these results, this represented substantial heterogeneity.

If the included studies were not clinically diverse and had similar outcome measures, a Meta - analysis was carried out in Review Manager software (RevMan 5) by one review author (AM). For continuous data, if heterogeneity was low, an inverse variance fixed-effect method was used. If heterogeneity was high, an inverse variance random-effects method was used. For dichotomous data, if heterogeneity was low, a Mantel-Haenszel fixed-effects method was used. If heterogeneity was high, a Mantel- Haenszel random-effects method was used. The source of heterogeneity was explored through subgroup analysis with respect to the type of intervention. If studies were too diverse, no Meta-analysis was conducted and a narrative synthesis was provided. We had intended to perform sensitivity analysis with respect to study quality in order to investigate the robustness of our findings but this could not be done mainly because most of the meta-analysis had too few studies (mostly two) to warrant sensitivity analysis. In some cases, all the studies in the meta-analysis had similar study quality thus rendering sensitivity analysis inappropriate.

## Results

### Results of the search and description of studies

**Figure 2:**
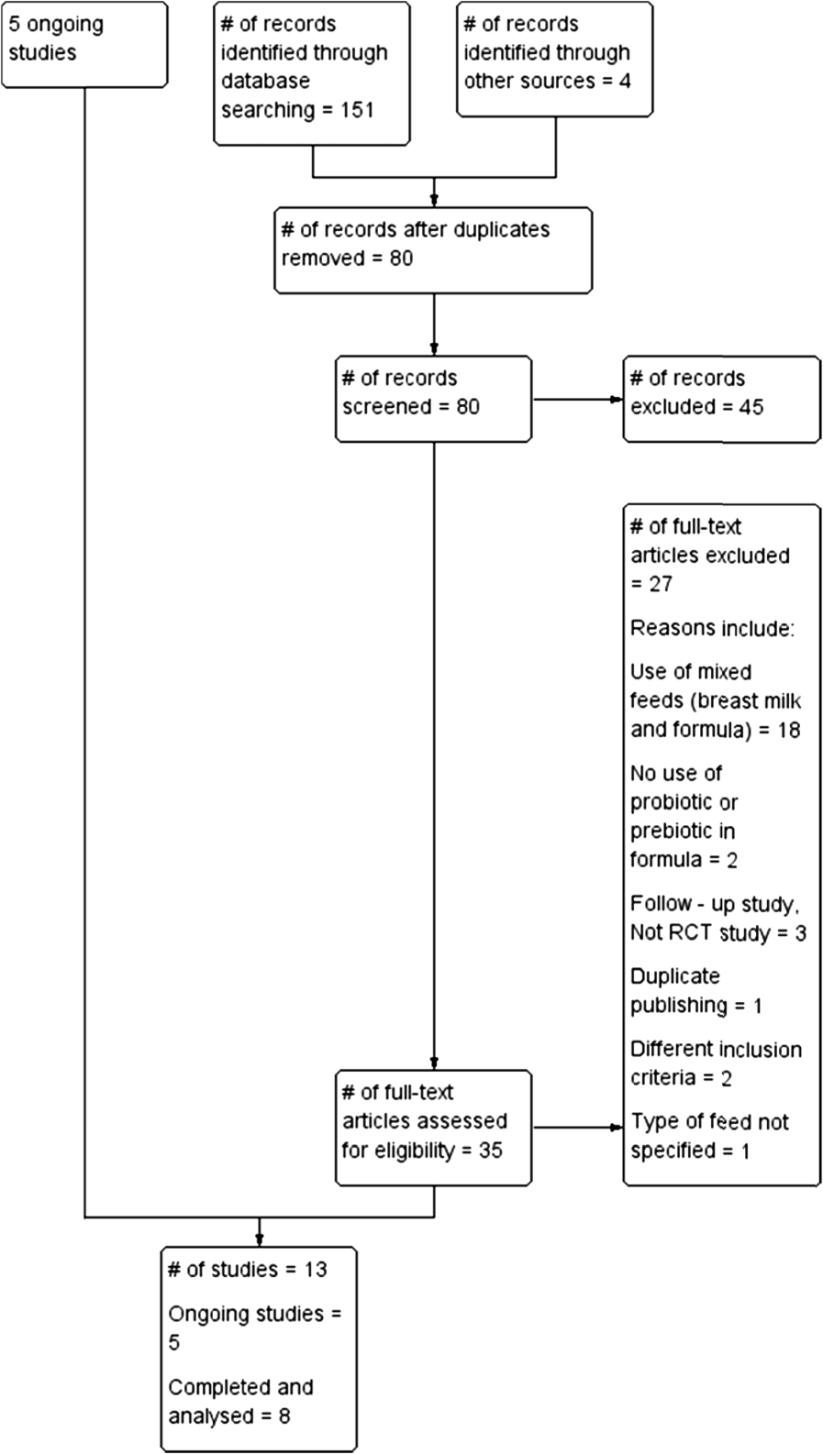
Process followed in the selection of studies.

Electronic search of available databases yielded 151 citations. After reading titles, abstracts, the duplicate reports were removed and 35 potentially relevant articles were identified. A hand search yielded 4 more articles. The full text reports were retrieved and reviewed for eligibility. One study was published in two other reports. The three studies were considered as one study since they reported the same identical study and are referred to as Boehm 2002 in this review [36-38]. Eight published studies (four probiotic and four prebiotic studies) [36,39-45] and five on-going studies were included in this review [46-51]. The process followed is shown in Figure 2. Table 2 gives a list of 27 studies which were excluded for: use of breast milk or mixed feeds (18 studies), no use of probiotic or prebiotic (2 studies), being a follow –up study, not RCT (3 studies), duplicate publishing (1 study); using different inclusion criteria with different outcomes (2 studies) and type of feed was unspecified (1 study). No eligible studies were excluded for failure to report the review’s pre-specified outcomes. 

**Table 2:** Excluded studies with reasons for exclusion

**Reasons for exclusion of studies**							
**Use of breast milk or mixed feeds (breast milk and formula)**			**Nouse of probiotic, prebiotic**	**Follow up - study, Not RCT**	**Duplicate publishing**	**Different inclusion criteria and outcomes**	**Type of feed unspecified**
Agarwal 2003 [52]	Lin H-C 2008 [53]	Riskin 2009 [54]	Andrews 1969 [55]	Chou I-C 2009 [56]	Stansbridge 1993 [57]	Cukrowska 2002 [58]	Karvonen 2002 [51]
Bin-Nun 2005 [59]	Manzoni 2006 [60]	Rouge 2009 [61]	Taylor 2009 [62]	Hoyos 1999 [63]		Wang 2007 [64]	
Dani 2002 [65]	Millar 1993 [66]	Samanta 2005 [67]		Lidesteri 2003 [68]			
Kitajima 1997 [69]	Mohan 2006 [70]	Westerbeek 2008 [71]					
Lee 2007 [72]	Mohan 2008 [73]	Westerbeek 2010 [74]					
Lin H-C 2005 [75]	Patole 2005 [76]	Yong Gu 2009 [77]					

A summary of the included probiotic, prebiotic and on-going studies are shown in Tables 3, 4 and 5. The included probiotic studies (N=212) were conducted in Greece, Italy and United States of America (USA). Treatment duration was 30 days using different probiotics. All four probiotic studies reported short term growth parameters (weight gain) which were recorded daily during the entire study duration [Table 3]. None of the probiotic studies reported data on: other types of infections, use of parenteral nutrition, feed intolerance (gastric aspirate [ml], abdominal distension) and stool consistency. The included prebiotic studies (N=126) were conducted in conducted in Greece, Italy, and Germany. Treatment duration ranged from 14 days to 28 days. All four prebiotic studies reported short term growth parameters (weight gain, length, head growth) which were recorded at different intervals during the entire study duration [Table 4]. None of the prebiotic studies reported data on: complications (NEC, sepsis, other types of infections, death / mortality), use of parenteral nutrition, feed intolerance (vomiting, gastric aspirate [ml], abdominal distension) and changes in intestinal permeability.

**Table 3:** A summary of four included probiotic studies

	**Costalos 2003 [**[39]	**Indrio 2008 [**[42]	**Reuman 1986 [**[41]	**Stratiki 2007 [**[40]
Location of study	Athens, Greece	University of Bari, Policinico, Italy	Gainesville, Florida, USA	Alexandra Regional Hospital, Greece
Participants - inclusion criteria	28 - 32 weeks gestation	3- 5 days old, appropriate for gestational age, preterm infants with normal agpar scores	Premature infants, <2000g at birth, less than 72 hours old (>24 old to <72 hours old)	27 to 37 weeks gestation, in stable state
Number of study participants	Study group=51 , Placebo = 36	Study group = 10 , Placebo = 10	Study group = 15, Placebo = 15	Study group = 41, Placebo = 34
Probiotic bacteria used	*Saccharomyces Bourlardii*	*Lactobacillus Reuteri ATCC 55730*	*Lactobacillus acidophilus*	*Bifidobacteriumlactis*
Dose of probiotic	10^9^cfu at 50mg/kg every 12 hours	1 X 10^8^cfu/day	9 X 10^6^cfu/ml formula	2 X 10^7^cfu/g milk powder
Placebo	Maltodextrin	Indistinguishable placebo	Conventional preterm formula	Conventional preterm formula
Dose of placebo	50 mg /kg / 12 hours	Not reported		
Treatment initiation	1st week of life as soon as enteral feed was tolerated	At 3–5 days of life	1st 72 hours of life	1st 2 days of life
Treatment duration	30 days	30 days	Not specified	30 days
**Reported Outcomes**				
Growth parameters	Weight gain	Weight gain	Weight gain	Weight gain, Linear growth, Head circumference
Timing and duration of measurement of growth parameters	Measured daily for 30 days	Measured daily for 30 days	Measured daily, duration not specified	Weight gain: measured daily, Lineargrowth (measured weekly), Head circumference (measured weekly)
Feed tolerance	Number of days to full enteral feed, Maximal enteral feed, vomiting	Number of days to full enteral feed, Maximal enteral feed, vomiting	Maximal enteral feed	Number of days to full enteral feed, Maximal enteral feed
Stool characteristics		Stooling frequency		
Complications	NEC, Sepsis		Mortality / death	NEC, Sepsis
Intestinal permeability	Changes in Intestinal permeability			Changes in Intestinal permeability
Changes in gastrointestinal microflora	cfu of bifidobacteria, lactobacillus, pathogens			cfu of bifidobacteria

**Table 4:** A summary of four included prebiotic studies

	**Boehm 2002 [**[36]	**Indrio 2009 [**[43]	**Kapiki 2007 [**[45]	**Mihatsch 2006 [**[44]
Location of study	Milan, Italy	University of Bari, Policinico, Italy	Athens, Greece	Ulm University, Germany
Participants - entry criteria	<32 weeks gestation	Healthy preterm newborns	≤ 36 weeks gestation	< 1500 g birth weight
Number of study participants	Study group = 15, Placebo = 15	Study group = 10 , Placebo = 10	Study group = 36, Placebo = 20	Study group = 10, Placebo = 10
Prebiotic used	GOS 90%, FOS 10%	scGOS, lcFOS at ratio 9:1	FOS	GOS, FOS
Dose of prebiotic	1g/dl	0.8 g/dl	0.4g/100ml	1g/dl
Placebo	Maltodextrin	Maltodextrin	Maltodextrin	Maltodextrin
Dose of placebo	1 g/dl	0.8 g/dl	0.4 g	1.8 / 90 ml
Treatment initiation	When enteral feed ≥ 80 mls /kg/day was tolerated	Not clear	Exclusively formula fed at start of study	At full enteral feed at start of study
Treatment duration	28 days	15 days	14 days	15 days
**Reported Outcomes**				
Growth parameters	Weight gain, linear growth	Weight gain, linear growth, head growth	Weight gain, linear growth, head growth	Weight gain
Timing and duration of measurement of growth parameters	Measured on days 1, 7, 14, 28	Measured before start of study, days 3, 5, 15	Measured on days 1, 7, 14	Weight gain: reported as “Average weight gain during study.”
Feed tolerance	Number of days to full enteral feed, maximal enteral feed	Number of days to full enteral feed, maximal enteral feed	Number of days to full enteral feed	Number of days to full enteral feed, maximal enteral feed
Stool characteristics	Stooling frequency, consistency		Stooling frequency, consistency	Stool viscosity, Stooling frequency, consistency
Changes in gastrointestinal microflora	cfu bifidobacteria		cfu bifidobacteria, pathogens	

**Table 5:** A summary of five on-going studies

	**Jacobs 2007 [**[46]	**Lozano 2008 [**[47]	**Al-Hosni 2010 [**[48]	**Patole 2009 [**[49]	**Underwood 2009 [**[50]
**Location of study**	Australia	Colombia	USA	Australia	USA
**Participants - inclusion criteria**	<32 weeks gestation, <1500 g birth weight, 1–3 days old	Birth weight <2000 grams, < 48 hours of age, admission in NICU, Hemodynamic-ally stable	Extremely Low Birth weight infants: < 1000 grams, 1 to 14 old, intention to start enteral feeds	32 weeks Gestation and 6 days, <1500g birth weight, ready to commence on enteral feeds for up to 12 hours	< 500grams birth weight, age less than 33 weeks gestation, exclusively formula fed
**Probiotic bacteria used**	*Bifidobacteriuminfantis, BifidobacteriumBifidus, Streptococcus thermophilus*	*Lactobacillus reuteri DSM 17938*	*Lactobacillus rhamnosus GG, Bifidobacteriuminfantis*	*Lactobacillus acidophilus 375 million, bifidobacteriumbifidum, bifidobacteria longus*	1. ProlactPlus
					2. GOS
					3*. Bifidobacteriuminfantis*
					4*. Bifidobacteriumanimalis*
**Dose**	1X10^9^	1X108 CFU in 5 drops of oil suspension 1/ day until discharge.	*L rhamnosus*: 500 million cfu, *B.infantis*: 500 million cfu	*L. acidophilus*:375 m organisms, *B bifidum, B. longus*: 125 million organisms	1. week 1 95:5 to week 5 75:25
					2. week: 0.25g/dL, to week 5: 2.0 g/dL
					3. week 1: 5X10^7^, to week 5: 4.2 X10^9^
					4. week 1: 5X10^7^, to week 5: 4.2 X10^9^
**Start date of study**	July- 2007	August 2008	February 2008	June 2009	June 2009
**Reported Outcomes**	Sepsis,	Sepsis	Average weight gain	Sepsis	Fecal microflora
	NEC	NEC	Growth velocity	NEC	
	Death	Death	Feed tolerance	All-cause mortality	
	Frequency of events		Volume of feed/day	Time to reach full feeds (150 mls/kg/day)	
	Length of hospital admission			Gut colonisation by probiotic	
	Number of antibiotic courses				
	Days to full enteral feeds				

### Risk of bias

**Figure 3:**
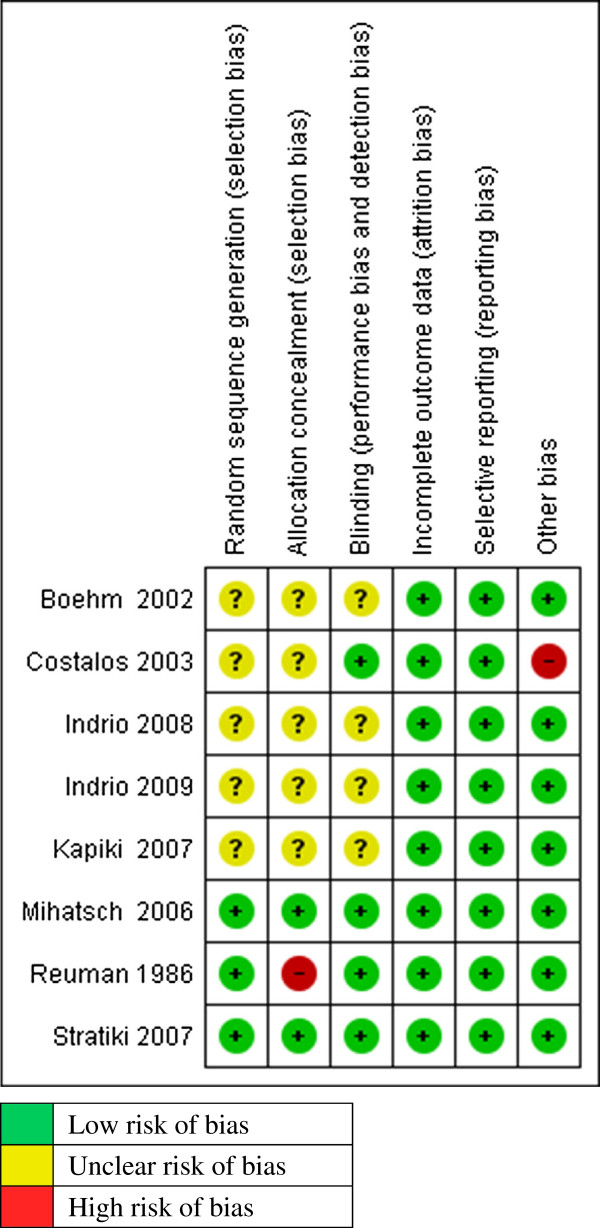
Methodological quality of included studies.

The quality of the included studies was assessed across six domains using guidelines from the Cochrane Handbook for Systematic Reviews of Interventions [35] (Figure 3). 

Random sequence generation: Three trials described clearly the methods used for random sequence generation [40,41,44]. Mihatsch used computer generated random lists with variable block sizes [44]. Stratiki used balance block randomization using random numbers [40] and Reuman used random numbers list combined with the last digit of the patients’ medical record [41]. The method used for random sequence generation was not clearly described 5 studies [36,39,42,43,45].

Allocation Concealment: In two trials treatment allocation was adequately concealed [33,40]. In the Stratiki trial, treatment allocation was conducted by a third party who was not involved in the study (Nutritional service) [40]. Mihatsch used precoded sachets in sealed envelopes [44]. In one study treatment allocation was not adequately concealed because the method used was alternation, matching of infants by birth weight and gestational age [41]. In the rest of the studies, allocation concealment was not clearly demonstrated or described [36,39,42,43].

Blinding: Blinding of study participants, care providers and assessors was clearly done in 4 trials [39-41,44]. In the other 4 trials, there was not enough information given on the blinding method to make a judgement [36,42,43,45].

Incomplete outcome data: Reported outcome data was satisfactory for all the eight included studies. Five studies had no missing outcome data [36,41-44]. In other three studies, the missing outcome data was balanced across the intervention groups with similar reasons reported [39,40,45].

Selective reporting (reporting bias): In all eight studies, the pre-specified outcomes in the methods section were reported in the results section [36,39-45].

Other potential sources of bias: Only one trial had a baseline imbalance which was a potential source of bias. Costalos had 51 infants enrolled in the treatment group and 36 infants in the placebo group. No explanation was presented whether the imbalance was due to a problem at randomization stage [39]. All other studies appeared to be free from other potential sources of bias.

## Effects of interventions

### Probiotics versus control

Four studies investigated the effect of probiotic administration versus no probiotic (control group) [39-42].

#### Primary outcomes: short term growth parameters

##### Weight gain

**Figure 4:**
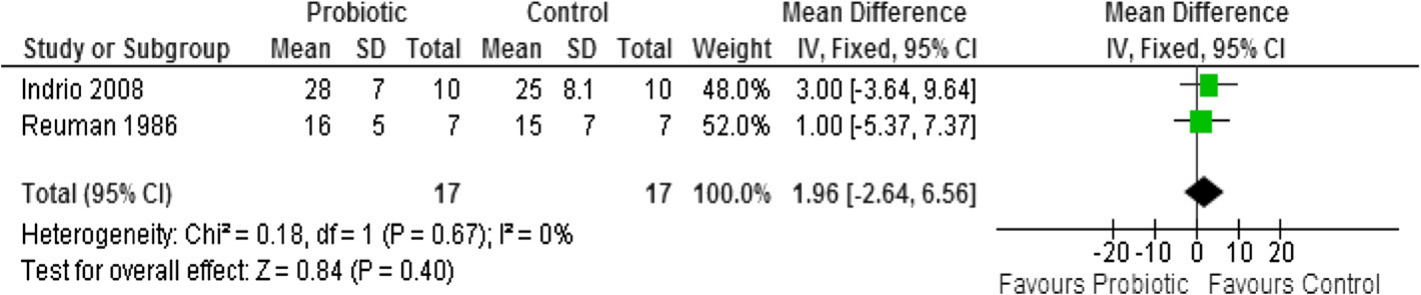
Effect of probiotic administration on weight gain (g/day).

All four studies reported on weight gain [39-42]. Results from two studies (n=34) were pooled in a meta-analysis [41,42]. There was no statistically significant difference in weight gain (g/day) between the probiotic and control groups (MD 1.96, 95% CI: -2.64 to 6.56). No statistically significant heterogeneity was observed (Chi^2^=0.18, p=0.67, I^2^=0%) (Figure 4) 

Two studies [39,40] reported their results using medians and could not be pooled in a meta - analysis. Costalos 2003 reported no statistically significant difference in weight gain (g/week) between the probiotic and control groups (p>0.05) [median (Interquartile range) of 163.5 (17.7) for the probiotic group (n=51) compared to 155.8 (16.5) for the control group (n=36)] [39]. Stratiki 2007 also reported no statistically significant difference in weight gain (g/day) between the probiotic and control groups (p=0.144) [median (range) of 28.3 (12 to 38) for the probiotic group (n=41) compared to 30 (10 to 40) for the control group (n=34)] [40].

##### Linear growth

Only one study reported this outcome but found no statistically significant difference in length gain (cm/week) between the probiotic and control groups (p=0.124) [median (range) of 1.4 (0 to 3) for the probiotic group (n=41) compared to 1.5 (0 to 3.5) for the control group (n=34)] [40].

##### Head growth

Only one study reported this outcome but found no statistically significant difference in head growth (cm/week) between the probiotic and control groups (p=0.124) [median (range) of 1.1 (0.45 to 1.9) for the probiotic group (n=41) compared to 0.9 (0 to 2) for the control group (n=34)] [40].

### Secondary outcomes

#### Complications

##### Necrotizing enterocolitis [NEC]

**Figure 5:**
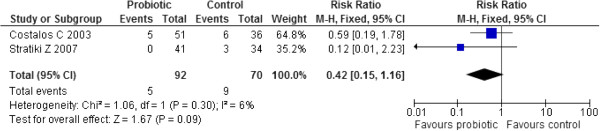
Effect of probiotic administration on NEC.

Two studies (n=162) reported on NEC and their results were pooled in a meta-analysis [39,40]. Administration of probiotics failed to significantly reduce the risk of NEC compared to controls (RR 0.42, 95% CI: 0.15 to 1.16). No significant heterogeneity was observed (Chi^2^=1.06, p=0.30, I^2^=6%) (Figure 5). 

##### Sepsis

**Figure 6:**
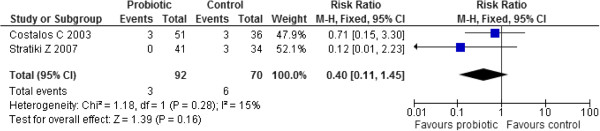
Effect of probiotic administration on sepsis.

Two studies (n=162) reported on sepsis and their results were pooled in a meta-analysis [39,40]. Administration of probiotics failed to significantly reduce the risk of sepsis compared to controls (RR 0.40, 95% CI: 0.11 to 1.45. No significant heterogeneity was observed (Chi^2^=1.18, p=0.28, I^2^=15%). (Figure 6) 

##### Other infections

No study reported on this outcome.

##### Mortality

Only one study [42] reported on mortality. The risk ratio for this one study (n=30) was calculated and it showed that the probiotics failed to significantly reduce the risk of death compared to the control (RR 0.33, 95% CI: 0.04 to 2.85).

##### Number of days on parenteral nutrition

No study reported on this outcome.

##### Number of days to full enteral feed

Two studies reported this outcome but their results could not be pooled in a meta-analysis because they reported the outcome in terms of medians and ranges [39,40]. Costalos 2003 reported no statistically significant difference in the number of days to full enteral feeding between the two groups (p>0.1) [median (IQR) of 9.3 (2.7) for the probiotic group (n=51) and 9.9 (4.5) for the control group (n=36)] [32]. Stratiki 2007 also reported no statistically significant difference in the number of days to full enteral feeding [median (range) of 10 (0 to 52) for the probiotic group (n=41) and 10 (0 to 30) for the control group (n=34)] [40].

##### Maximal enteral feed

All four studies reported on this outcome [39-42]. Results from two studies (n=34) were pooled in a meta-analysis as they both reported the average amount of feeding (ml/day) in terms of mean (SD) [41,42]. There was no statistically significant difference in the mean amount of feeding (ml/day) between the probiotic and control groups (MD 35.20, 95% CI: -7.61 to 78.02) No statistically significant heterogeneity was observed between the studies (Chi^2^=1.65, p=0.20, I^2^=39%).

Costalos 2003 reported no statistically significant difference in the milk intake (ml/kg/day) at maximal enteral feeding (p>0.1) [median (IQR) of 155 (15) for the probiotic group (n=51) versus 148 (13) for the control group (n=36)] [39]. Stratiki 2007 also reported no statistically significant difference in the maximal milk intake (ml/kg/day) (p=0.624) [median (range) of 210 (165 to 250) for the probiotic (n=41) group versus 192 (120 to 250) for the control group (n=34)] [40].

##### Feed tolerance: vomiting, gastric aspirate, abdominal distension

Two studies (n=107) reported on vomiting and were pooled in a meta-analysis [39,42]. There was no statistically significant difference in the frequency of vomiting between the probiotic and control groups (RR 0.78, 95% CI: 0.18 to 3.37). No statistically significant heterogeneity was observed (Chi^2^=0.41, p=0.52, I^2^=0%).

In all four probiotic studies, there were no reported incidences of gastric aspirates, abdominal distension or diarrhea. Authors were further contacted for clarification and one responded [42] and stated categorically that none of these symptoms were observed.

#### Stool characteristics

##### Stool frequency

Only one study (n=20) reported stool frequency as the number of episodes of evacuations per day in terms of mean (SD) [42]. The mean difference for this one study was calculated and it showed that probiotic consumption resulted in a statistically significant larger number of stools per day compared to the control group (MD 1.60, 95% CI: 1.20 to 2.00).

##### Stool consistency

No study reported on the effects of probiotics on stool consistency.

##### Changes in intestinal permeability

Two studies reported this outcome but their results could not be pooled in a meta-analysis [39,40]. The studies used two different tests to test for intestinal permeability. Costalos 2003 used a 1-hour D-Xylose blood test and reported no statistically significant difference between the two groups (p>0.1) [median (IQR) of 1.5 (0.4) millimols/L for the probiotics (n=51) and 1.35 (0.3) mmol/L for the control (n=36)] [39]. Stratiki 2007 used a lactulose/mannitol (L/M) urine test and reported no statistically significant difference in the L/M ratios between the probiotic and control groups (p=0.073) but the values for median (range) were presented in a figure from which they could not be accurately extracted [40].

#### Changes in gastrointestinal micro flora

##### Bifidobacteria

Two studies reported on bifidobacteria but their results could not be pooled in a meta-analysis [39,40]. Costalos 2003 reported a significantly higher log viable Bifidobacteria counts per gram of positive infants in the probiotics group compared to the controls (p<0.001) [median (IQR) of 2.65 (0.083) for the probiotics group (n=51) and 2.27 (0.075) for the control group (n=36)] [39]. Stratiki 2007 reported bifidobacteria in terms of log 10 cfu/g wet feces but found no statistically significant difference between the two groups (p=0.075) [median (range) of 9.7 (7.5-10.3) for the probiotics group (n=41) and 8.9 (7.2-10.2) for the control group (n=34)] [40].

##### Lactobacillus

Only one study reported on lactobacillus [39]. This study reported no statistically significant difference in the log viable bacterial lactobacillus counts per gram of positive infants between the two groups (p>0.05) [median (IQR) of 1.57 (0.285) for the probiotics group (n=51) and 1.42 (0.287) for the control group (n=36)].

##### Pathogens

Only one study reported this outcome (enterococci, bacteroides, and staphylococci) in terms of the median (IQR) of log viable bacterial counts per gram of positive infants [39] (Table 6). The study reported significantly higher counts of Enterococci (p<0.05) and Staphylococci (p<0.001) in the probiotic group compared to the controls. However, the study found no statistically significant difference in the counts of bacteroides between the two groups (p>0.05). 

**Table 6:** Log viable bacteria counts per gram of stool in positive infants fed probiotics

**Costalos 2003**[39]	**Median (IQR)**	
**Pathogens**	**Probiotic**	**Control**
	**n= 51**	**n=36**
Enterococci	2.14 (0.359)	2.19 (0.138)
Bacteriodes	2.17 (0.164)	2.25 (0.363)
Staphylococci	1.23 (0.869)	0.6 (0.281)

### Prebiotic versus control

Four studies investigated the effect of prebiotics administration versus no prebiotics (control group) [36,43-45].

#### Primary outcomes: short-term growth parameters

##### Weight gain

**Figure 7:**
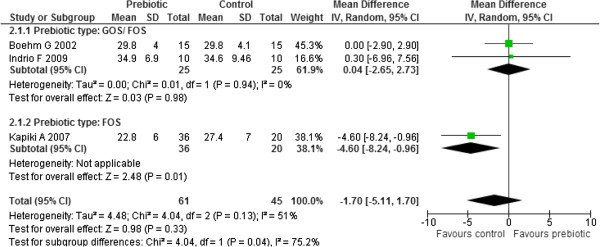
Effect of prebiotic administration of weight gain (g/day).

All four studies reported on weight gain [36,43-45]. Results from three studies (n=106) were pooled in a meta-analysis [36,43,45]. Moderate heterogeneity was observed between the studies (Chi^2^=4.04, p=0.13, I^2^=51%). An investigation of heterogeneity by subgroup analysis with respect to the prebiotic type used (GOS/ FOS versus FOS only) yielded statistically significant subgroup differences (Chi^2^=4.04, df=1, p=0.04, I^2^=75.2%) implying that prebiotic type may be the source of heterogeneity. There was no statistically significant heterogeneity between the two studies in the GOS/ FOS subgroup (Chi^2^=0.01, df=1, p=0.94, I^2^=0%) [36,43]. The results for the GOS/FOS subgroup yielded no significant difference in weight gain (g/ day) between the two groups (MD 0.04, 95% CI: -2.65 to 2.73, n=50, 2 studies) while the other FOS subgroup yielded a significantly higher weight gain in controls compared to the prebiotics (MD −4.60, 95% CI: -8.24 to −0.96, n=56, 1 study). (Figure 7) Sensitivity analysis with respect to study quality could not be done because all three studies were of poor quality since the methods used for sequence generation, allocation concealment and blinding were all not clear. 

Mihatsch 2006 reported no statistically significant difference in weight gain (g/kg/day) between the two groups (p=0.4) [median (range) of 17.6 (8.1 to 23.4) for the prebiotic group (n=10) compared to 13 (9.3 to 21.9) for the control group (n=10)] [44].

##### Linear growth

**Figure 8:**
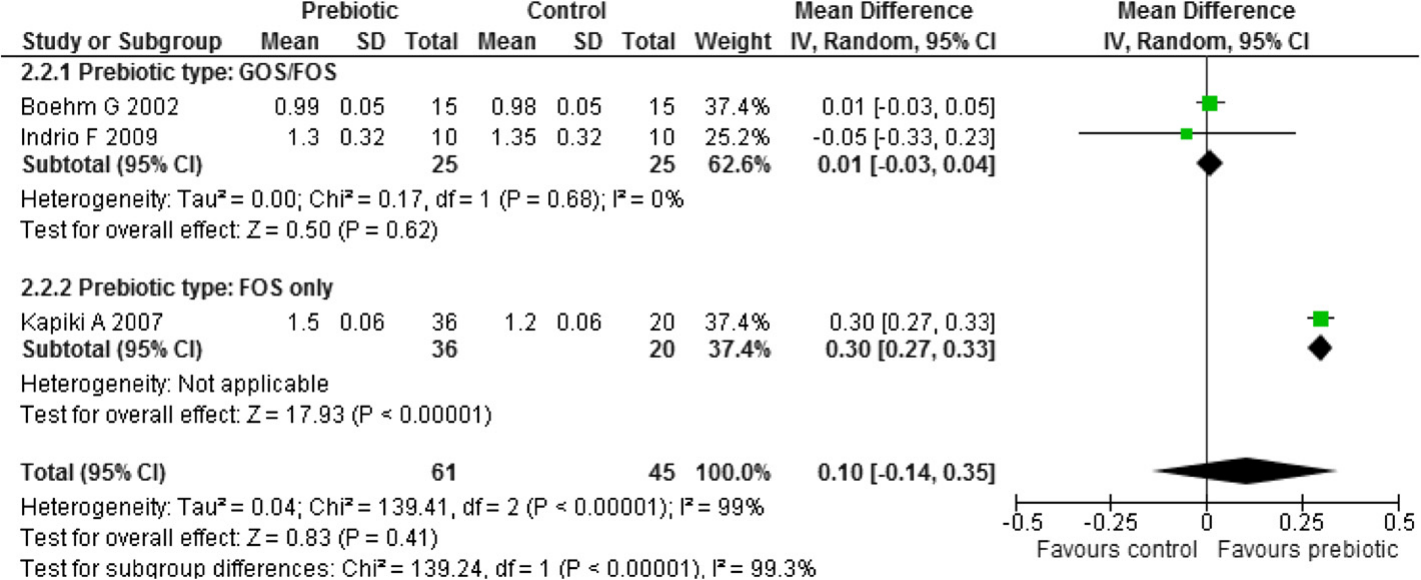
Effect of prebiotic administration of linear growth (cm/week).

Three studies reported on length gain [36,43,45]. Meta-analysis of the results from these three studies (n=106) revealed significant heterogeneity between the three studies (Chi^2^ = 139.41, df = 2, p < 0.00001, I^2^ = 99%). An investigation of heterogeneity by subgroup analysis with respect to the prebiotic type used (GOS/ FOS versus FOS only) yielded statistically significant subgroup differences (Chi^2^=139.41, df=1, p<0.00001, I^2^=0%) implying that prebiotic type may be the source of heterogeneity. There was no statistically significant heterogeneity between the two studies in the GOS/ FOS subgroup (Chi^2^=0.17, df=1, p=0.68, I^2^=0%). [36,43]. The results for the GOS/FOS subgroup yielded no statistically significant difference in length gain (cm/ week) between the two groups (MD 0.01, 95% CI: -0.03 to 0.04, n=50, 2 studies) while the other FOS subgroup yielded a significantly higher length gain (cm/ week) in prebiotics compared to the controls (MD 0.30, 95% CI: 0.27 to 0.33, n=56, 1 study). (Figure 8) Sensitivity analysis with respect to study quality could not be done because all three studies were of poor quality since the methods used for sequence generation, allocation concealment and blinding were all not clear. 

##### Head growth

Two studies reported on head growth (cm/week) [43,45]. Meta-analysis of the results from these two studies (n=76) failed to yield statistically significant difference in head growth (MD −0.01, 95% CI: -0.02 to 0.00). No significant heterogeneity was detected between the two studies (Chi^2^ = 0.10, p =0.75, I^2^ = 0%).

#### Secondary outcomes

##### Complications

No prebiotic study reported on Necrotizing Enterocolitis (NEC), Sepsis, other infections and mortality.

#### Feeding tolerance

##### Number of days on parenteral nutrition

No study reported on parenteral nutrition.

##### Age at full enteral feed

Two studies reported on age at full enteral feeds [36,45]. Meta-analysis of the results from these two studies (n=86) did not find statistically significant difference in the age at full enteral feed (MD −0.79, 95% CI: -2.20 to 0.61). No significant heterogeneity was detected between the two studies (Chi^2^ =1.16, p =0.28, I^2^ = 14%).

##### Maximal enteral feed

Two studies reported on this outcome but their results could not be pooled in a meta-analysis [36,44]. Boehm 2002 reported the feeding volume (ml/kg/day) in terms of the mean (SD) and therefore a mean difference was calculated. There was no statistically significant difference in feeding volume between the prebiotics group (n=15) and control groups (n=15) (MD −4.10, 95% CI: -18.16 to 9.96) [36].

Mihatsch 2006 reported no statistically significant difference in the average formula intake within the study period (ml/kg/d) between the two groups (p=0.35) [median (range) of 156 (127 to 165) for the prebiotic group (n=10) compared to 151 (117 to 169) for the control group (n=10)] [44].

##### Feed tolerance: vomiting, gastric aspirate, abdominal distension, diarrhea

All four studies reported this outcome [36,43-45]. In all 4 studies (n=126), there were no observed incidences of feed intolerance. There was no vomiting, gastric aspirate removed, no abdominal distension or diarrhea reported. All infants tolerated the preterm formula with prebiotic or control. From further communication with study authors, 2 study authors [43,44] responded that none of these outcomes were observed.

#### Stool characteristics

##### Stool frequency

**Figure 9:**
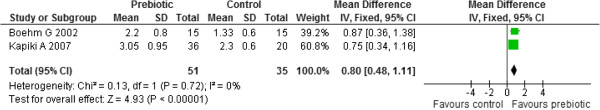
Effect of prebiotic administration on stool frequency.

Three studies reported on stool frequency [36,44,45]. Two studies reported the results in form of mean (SD) of the number of stools per day (number/ day) [36,45]. Meta-analysis of results from these two studies (n=86) showed a significantly higher stool frequency in the prebiotic group compared to the control group (MD 0.80, 95% CI: 0.48 to 1.1). No significant heterogeneity was detected between the two studies (Chi^2^ =0.13, p =0.72, I^2^ = 0%) (Figure 9). 

Mihatsch 2006 reported no statistically significant difference in stool frequency between the two groups (p=0.059) [median (range) of 3.6(1.7 to 6.9) stools/day in prebiotic group (n=10) compared to 2.6 (2 to 4.9) stools/day in control group (n=10)] [44].

##### Stool consistency

Three studies reported on stool consistency but using three different scales of measurement [36,44,45]. Although two studies [36,45] both measured consistency in form of a scale ranging from 1 to 5 and reported their results as mean (SD), they could not be pooled in a meta-analysis because their scales were going in opposite directions; Boehm 2002 (1=watery, 2=soft, 3=seedy, 4=formed, 5=hard) [36]. Kapiki 2007 (5=watery, 4=loose, 3=soft, 2=firm, hard=1) [45]. The mean differences for these two studies were therefore calculated separately.

In Boehm 2002, the stools from the prebiotic group (n=15) were significantly more watery as compared to the control group (n=15). (MD −0.91, 95% CI: -1.41 to −0.37) [36]. In Kapiki 2007, the stools from the prebiotic group (n=36) were significantly harder as compared to the control group (n=20). (MD −0.34, 95% CI: -0.66 to −0.02) [45].

Mihatsch 2006 reported a statistically significantly lower stool viscosity at day 14 (Newtons) for the prebiotics compared to controls (p=0.006) [median (range) of 31.8 (1.9 to 67.3) in the prebiotic group (n=10) compared to 157.5 (24.1 to 314.0) in the control group (n=10)] [44].

#### Changes in intestinal permeability

No prebiotic study reported on changes in intestinal permeability.

#### Changes in gastrointestinal micro flora

##### Bifidobacteria

**Figure 10:**
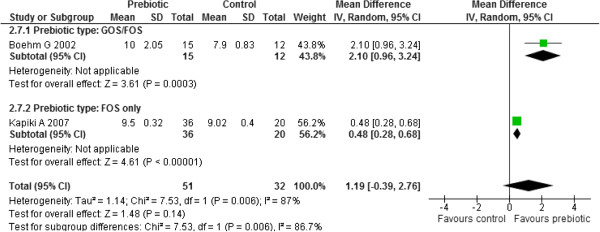
Effect of prebiotic administration on total counts of Bifidobacteria.

Two studies reported on this outcome [36,45]. Meta-analysis of these two studies (n=84) revealed statistically significant heterogeneity between the two studies (Chi^2^ =7.63, p =0.006, I^2^ = 87%). An investigation of heterogeneity by subgroup analysis with respect to the prebiotic type used (GOS/ FOS versus FOS only) yielded statistically significant subgroup differences (Chi^2^ =7.63, p =0.006, I^2^ = 86.7%) implying that prebiotic type may be the source of heterogeneity. The results for the GOS/FOS subgroup yielded significantly higher bifidobacteria counts in prebiotics compared to controls (MD 2.10, 95% CI: 0.96 to 3.24) [36]. The other FOS subgroup also yielded significantly higher bifidobacteria counts in prebiotics compared to controls (MD 0.48, 95% CI: 0.28 to 0.68) [45] (Figure 10). 

##### Lactobacilli

Only one study [36] reported this outcome but the actual values were not given.

##### Pathogens [Post-intervention]

Two studies reported on this but their results could not be pooled in a meta-analysis [36,45]. Boehm 2002 reported the sum of clinically relevant pathogens at the end of the intervention period in the form of mean (SD) log cfu/g stool. The values were used to calculate the mean difference which showed that the sum of the studied pathogens was significantly lower in the prebiotic group (n=12) compared to the control group (n=13). (MD −0.43, 95% CI: -0.79 to −0.07) [36].

Kapiki 2007 reported this outcome (staphylococci, E. coli, bacteroides, and enterococci) in terms of mean (SD) log 10 CFU/g wet feces [45]. Mean differences for each of these pathogens were calculated. There was no statistically significant difference in the number of staphylococci (MD 0.00, 95% CI: -0.17 to 0.17) between the two groups but there were significantly fewer E. coli (MD −1.69, 95% CI: -1.85 to −1.53) and enterococci (MD −0.80, 95% CI: -0.99 to −0.61) in the prebiotic group (n=36) compared to the control group (n=20). With regards to bacteroides, there were significantly more bacteroides in the prebiotic group (n=36) compared to the control group (n=20) (MD 0.50, 95% CI: 0.36 to 0.64) [45].

## Discussion

The objective of this review was to assess if addition of probiotics or prebiotics to preterm infant formula led to improved growth and clinical outcomes in preterm or low birth weight infants. Studies that used breast milk or mixed feeds (breast milk and infant formula) were excluded. All RCTs evaluated probiotics or prebiotic use in preterm infants, were of small sample size, varied in enrolment criteria, intervention, treatment initiation and duration.

### Summary of main findings

#### Probiotics

This review was under powered to detect clinically important differences in primary outcomes (weight gain, linear growth, head growth) because of the few number of studies, small sample size (n=34) and poor methodological quality of studies. This review found no significant effect on weight gain from use of probiotics added to infant formula. There was also no significant probiotic effect on linear and head growth from the one study measuring these two outcomes. Probiotic supplementation failed to significantly reduce the risk of complications such as NEC, sepsis and death compared to control group. Outcomes such as number of days on parenteral nutrition and other infections were not reported. There was no significant difference in the amount of feed volume (ml/day) and frequency of vomiting between study groups. Preterm infant formula with probiotics was well tolerated as no gastric aspirates, abdominal distension or diarrhea was reported. Effects of probiotics on stool characteristics were under reported. Results from one study showed probiotics supplementation did result in a larger number of stools per day.

Effects on intestinal permeability could not be evaluated since two different laboratory tests (lactulose / mannitol ratio and D- xylose tests) were reported and the results could not be pooled. Sugar absorption tests (such as lactulose / mannitol ratio) are a direct measure of intestine integrity which reflects gut maturation and in research; they demonstrate the effects of experimental therapy [78,79]. Monitoring changes in intestinal permeability in preterm infants is essential since there is evidence that initiation of enteral feeds decreases intestinal permeability [78,80]. However, this could not be established in this review. Other outcomes such as age at full enteral feeds and intestinal micro flora (pathogens) could not be evaluated as medians (inter quartile ranges) were reported. No probiotic study reported any data on low birth weight infants therefore no conclusions could be made on this population.

The included probiotic studies had short treatment duration of 30 days. This confirms the European Society for Pediatric, Gastroenterology, Hepatology and Nutrition (ESPGHAN) statement that there is a “lack of published evidence on clinical benefits from long term use of probiotic containing infant formula” [81]. This review confirms that there is a need for long term follow-up RCTs on preterm infants. Live probiotic bacteria were used in the trials. There have been few reports of bacteraemia from probiotic use in the biomedical literature [82-84]. There were no cases of sepsis reported as a result of probiotic consumption in the included studies. In recent reviews, the time to reach full enteral feeds was earlier in the preterm infants given probiotics with breast milk or mixed feeds. This review could not evaluate this outcome. Well-designed RCTs with similar feeding regimes are needed to evaluate this outcome.

#### Prebiotics

This review was under powered to detect clinically important differences in primary outcomes (weight gain, linear growth, head growth) because of few number of studies, small sample size (n=106) and poor methodological quality of studies. Addition of prebiotic combinations of GOS /FOS or FOS alone to preterm infant formula did not have any significant effect on weight gain. Addition of GOS / FOS to preterm infant formula did not have any effect on linear growth. However, addition of FOS alone did have a significant effect on linear growth. Neither GOS / FOS combination nor FOS alone had any effect on head growth.

None of the prebiotic studies reported on NEC, sepsis, other infections, mortality (death), parenteral nutrition or changes in intestinal permeability; therefore these outcomes could not be evaluated. Prebiotics did not have any significant effect on the age at which infants reached full enteral feeds or volume of feed tolerated. Prebiotic preterm formula was well tolerated because there were no reports of vomiting, gastric aspirates, abdominal distension or diarrhea. Prebiotic supplementation did result in a higher stooling frequency compared to control. Effects on stool consistency were inconclusive as results from one study resulted in more watery stools in the prebiotic study group compared to control group, in a second study, the prebiotic group experienced harder stools compared to control group. The third study results were presented in medians (range) therefore no conclusions could be made. In preterm infants, frequent watery stools may signify intolerance, a transient lactase deficiency or another pathological state which always require further investigation [6].

Prebiotics did have a significant effect on intestinal micro flora. Addition of GOS / FOS combination or FOS alone significantly increased counts of bifidobacteria. Effects on lactobacillus counts could not be evaluated as actual figures were not available. The sum of studied pathogens and some selected pathogens (E- coli, enterococci) were significantly fewer in the prebiotic group compared to control group. There was no effect on staphylococci levels while bacteroides were significantly higher in the probiotic group compared to control group. No prebiotic study reported any data on low birth weight infants; therefore no evaluations could be made.

The prebiotic studies were of short duration ranging from 14 to 28 days. The dose of the prebiotic used (GOS, FOS) varied from 0.4 g/dl o 1g/dl. The European Committee on Food recommends that prebiotics added to formula milk do not exceed 0.8 g/100 ml. The rationale for prebiotic doses not exceeding 1g/ml in clinical trials is an attempt to maximize the bifidogenic effect with minimal intolerance as exhibited by, abdominal distension [85]. The preterm infants tolerated the prebiotic formula as there were no symptoms of feed intolerance reported.

Prebiotic supplementation did have some short term benefits: increased stooling frequency and bifidobacteria counts, fewer pathogens in the prebiotic group compared to control group. However, large RCTS with long term follow -up are needed to find out if these short term benefits translate into improved general health and reduced morbidities in preterm infants. Due to the short duration of prebiotic studies, routine supplementation with prebiotics in preterm infants cannot be recommended.

#### Quality of the evidence and potential biases

In this review, the quality of the evidence was compromised by several factors: Sample size: included studies were of small individual sample size, number of study participants ranged from 20 to 87 in the probiotic studies, 20 to 56 in prebiotic studies. Intervention: Different types of probiotic and prebiotics, doses and treatment duration were used. Methodological quality: Inadequate information was published to assess methodological quality of the studies. Information was missing on sequence generation, allocation concealment, blinding, incomplete outcome data, selective reporting and free of other bias domains. The significance of any relationship between methodological quality and study outcomes could not be verified since no subgroup analysis with respect to study quality could be done as a result of either too few studies in a meta-analysis or having all studies with similar quality in a meta-analysis. Not all the reviews pre- specified outcomes were addressed by the included studies.

At the conclusion of the review process and preparation of the manuscript (for this review), one on- going study was terminated due to being under powered [47]. One study was completed and data analysis commenced. The results from this study could not be included in this review [48]. The other three studies were still on-going [46,49,50]. The reviewers used thorough comprehensive search strategies adopted for the available databases. All attempts were made to minimize publication bias. All steps of this review were conducted independently by the reviewers.

#### Agreements and disagreements with other reviews

No significant difference was found in contrast with past reviews and that the potential reasons are lack of power, poor quality of studies or a lack of effect in formula fed infants. This review did agree with some aspects of past reviews. Prebiotics did have an impact on GI micro flora (increased bifidobacteria counts, reduction in certain pathogens); feed tolerance (no reported gastric aspirates, abdominal distension).

## Conclusion

There is not enough evidence to state that supplementation of preterm infant formula with probiotics or prebiotics does result in improved growth and clinical outcomes in preterm infants. Therefore this review does not support the routine supplementation of preterm formula with probiotics or prebiotics.

### Implications for research

For clear recommendations to be made, long term large RCTs on exclusively formula fed preterm and low birth weight infants are required to investigate the effects of probiotics and prebiotics supplementation in preventing NEC, sepsis, death/mortality; changes in intestinal micro flora and intestinal permeability; explore the efficacy of different doses of the same probiotic on clinical outcomes because available studies used different probiotic doses; similarly, explore the efficacy of different doses of the same prebiotic on clinical outcomes because available studies used similar prebiotics with different doses and treatment duration.

## Abbreviations

Cfu: Colony forming units; CI: Confidence interval; cm: Centimetres; ESPGHAN: European society for pediatric gastroenterology: hepatology and nutrition; FOS: Fructo-oligosaccharide; GI: Gastrointestinal; GOS: Galacto-oligosaccharide; IQR: Inter quartile range; IFN-γ: Interferon – gamma; IL-6: Interleukin – 6; IL-10: Interleukin – 10; IL-1β: Interleukin – 1beta; kg: Kilogram; L/M: Lactulose mannitol; MD: Mean difference mmol: millimols; ml: Millilitres; NEC: Necrotizing enterocolitis; TNF-α: Tissue necrosis factor – alpha; RCTs: Randomized controlled trials; RR: Risk ratio; SD: Standard deviation; USA: United States of America; WHO: World Health Organisation.

## Competing interests

The authors declared that they have no competing interests.

## Authors’ contributions

The authors contributed the following: MM: Developed review protocol, selected RCTs, carried out data extraction; assessment of risk of bias in included studies, developed, edited and critically reviewed the manuscript. ML: Selected RCTs, carried out data extraction, assessment of risk of bias in included studies, critically reviewed the manuscript. AM: Carried out the statistical analysis, interpretation of results and critically reviewed the manuscript. TY: Assisted in designing the review and critically reviewed the manuscript. RB: Assisted in designing the review and critically reviewed the manuscript. All authors read and approved the final manuscript.

## References

[B1] Anderson DM Samour PQ, Helm KK From Pediatric Nutrition Handbook of Pediatric Nutrition 2005 3 James and Bartlett Publishers, Sudbury, Massachusetts 53 71

[B2] Lissauer T Clayden G Neonatal Medicine Illustrated Text book of Pediatrics 2007 3 Elsevier, Mosby 145 168

[B3] Georgieff MK MacDonald MG, Seshia MMK, Mullet MD Nutrition Avery’s Neonatology pathophysiology and management of the new born 2005 6 Lippincott Williams and Wilkins, Philadelphia 380 381

[B4] Lissauer T Fanaroff A The preterm infant: growth and nutrition. In Neonatology at a glance 2006 Blackwell Publishing, Malden, Mass 76 77

[B5] Uhing MR Das UG Optimizing growth in the preterm infant Clin Perinatol 2009 36 165 176 10.1016/j.clp.2008.09.010 19161873

[B6] Anderson MS Johnson CB Townsend SF Hay W Merenstein GB, Gardner SL Enteral nutrition Handbook of neonatal intensive care 2002 5 Mosby, Mosby 314 316

[B7] Corpeleijin WE van Vliet I de Gast-Bakker DH van der Schoor SRD Alles MS Hoijer M Tibboel D van Goudoever JB Effect of enteral IGF-1 supplementation on feeding tolerance, growth and gut permeability in enterally fed premature neonates J Pediatr Gastrotenterol Nutr 2008 46 184 190 10.1097/MPG.0b013e31815affec 18223378

[B8] Chauhan M Henderson GM McGuire W Enteral feeding for very low birth weight infants: reducing the risk of necrotising enterocolitis Arch Dis Child Fetal Neonatal Ed 2008 93 F162 F166 18006565 10.1136/adc.2007.115824

[B9] Underwood MA Salzmand NH Bennett SH Barman M Mills DA Marcobal A Tancredi DJ Bevins CL Sherman M A randomized placebo - controlled comparison of 2 prebiotic/probiotic combinations in preterm infants: Impact on weight gain, intestinal microbiota and fecal short chain fatty acids J Pediatr Gastroenterol Nutr 2009 48 216 225 10.1097/MPG.0b013e31818de195 19179885 PMC2743418

[B10] Shah NP Functional cultures and health benefits Int Dairy J 2007 17 1262 1277 10.1016/j.idairyj.2007.01.014

[B11] Parvez S Malik KA Kang SA Kim HY Probiotics and their fermented food products are beneficial for health J Appl Microbiol 2006 100 1171 1185 10.1111/j.1365-2672.2006.02963.x 16696665

[B12] Gibson GR Nathalie D Inulin and oligofructose. New Scientific Developments Nutr Today 2008 43 54 59 10.1097/01.NT.0000303311.36663.39

[B13] Gibson GR Fibre and effects on probiotics (the prebiotic concept) Clin Nutr 2004 1 2 25 31

[B14] Macfarlane GT Steed H Macfarlane S Bacterial metabolism and health related effects of galacto-oligosaccharides and other prebiotics J Appl Microbiol 2008 104 305 344 18215222 10.1111/j.1365-2672.2007.03520.x

[B15] Losada M Olleros T Towards a healthier diet for the colon: the influence of fructooligosaccharides and lactobacilli on intestinal health Nutr Res 2002 22 71 84 10.1016/S0271-5317(01)00395-5

[B16] Watzl B Girrbach S Monika R Inulin, Oligofructose and immunomodulation Br J Nutr 2005 93 Suppl 1 S49 S55 15877895 10.1079/bjn20041357

[B17] FAO/WHO Guidelines for evaluation of probiotics in food, 2002 http/www.who.int/foodsafety/fs_management/en/probiotic_guidelines.pdf

[B18] Gill HS Probiotics enhance anti- effective defences in the gastrointestinal tract Best Pract Res Clin Gastroenterology 2003 17 755 773 10.1016/S1521-6918(03)00074-X 14507586

[B19] Schlee M Harder J Koten B Stange EF Wehkamp J Fellermann K Probiotic lactobacilli and VSL#3 induce enterocyte beta-defensin 2 Clin Exp Immunol 2008 151 528 535 10.1111/j.1365-2249.2007.03587.x 18190603 PMC2276967

[B20] Fedorak RN Madsen KK Probiotics and prebiotics in gastrointestinal disorders Curr Opin Gastroenterol 2004 20 146 155 10.1097/00001574-200403000-00017 15703637

[B21] Resta-Lenert S Barrett KE Live probiotics protect intestinal epithelial cells from the effects of infection with entero invasive Escherichia Coli (EIEC) Gut 2003 52 988 997 10.1136/gut.52.7.988 12801956 PMC1773702

[B22] Heyman M Terpend K Menard S Effects of specific lactic acid bacteria on the intestinal permeability to macromolecules and the inflammatory condition Acta Pediatr 2005 94 Suppl 449 34 36 10.1111/j.1651-2227.2005.tb02153.x 16214764

[B23] Boirvant M Strober W The mechanism of action of probiotics Curr Opin Gastroenterol 2007 23 670 692 10.1097/MOG.0b013e3282f0cffc 17906447

[B24] Olivares M Diaz-Ropero MP Gómez N Lara-Villoslada F Sierra S Maldonado JA Martin R Lopez-Huetas E Rodriguez JM Xaus J Oral administration of two probiotic strains Lactobacillus gasseri CECT5714 and Lactobacillus coryniformis CECT5711, enhances the intestinal function of healthy adults Int J Food Microbiol 2006 107 104 111 10.1016/j.ijfoodmicro.2005.08.019 16271414

[B25] Wult M Hagslatt MLJ Odenholt I Berggren A Lactobacillus planatarum 299v enhances the concentrations of fecal short chain fatty acids in patients with recurrent clostridium difficile associated diarrhea Dig Dis Sci 2007 52 2082 2086 10.1007/s10620-006-9123-3 17420953

[B26] Calame W Weseler AR Viebke C Flynn C Siemensma AD Gum Arabic establishes prebiotics functionality in healthy human volunteers in a dose dependent manner Br J Nutr 2008 100 1269 1275 10.1017/S0007114508981447 18466655

[B27] Cherbut C Michel C Raison V Kravtchenko T Severine M Acacia gum is a bifidogenic dietary fibre with high digestive tolerance in healthy humans Microb Ecol Health Dis 2003 15 43 50 10.1080/08910600310014377

[B28] Guarner F Studies with Inulin type fructans on intestinal infections, permeability and inflammation J Nutr 2007 137 2568S 2571S 17951504 10.1093/jn/137.11.2568S

[B29] Vlieger AM Robroch A van Buuren S Kiers J Rijkers G Benninga MA Tebiesebeke R Tolerance and safety of lactobacillus paracasei ssp paracasei in combination with Bifidobacterium animalis ssp lactis in a prebiotic-containing infant formula: a randomised controlled trial Br J Nutr 2009 102 869 875 10.1017/S0007114509289069 19331702

[B30] Kullen MJ Bettler J The delivery of probiotics and prebiotic to infants Curr Pharm Des 2005 11 55 74 10.2174/1381612053382359 15638752

[B31] AlFaleh K Anabrees J Bassler D Al-Kharfi T Probiotics for prevention of necrotizing enterocolitis in preterm infants Cochrane Database Sys Rev 2011 Art. No.: CD005496 Issue 3 10.1002/14651858.CD005496.pub3 21412889

[B32] Barclay AR Stenson B Simpson JH Lawrence T Wilson D Probiotics for Necrotizing Enterocolitis: A Systematic Review J Pediatr Gastroenterol Nutr 2007 45 569 576 10.1097/MPG.0b013e3181344694 18030235

[B33] Deshpande G Rao S Patole S Probiotics for prevention of necrotising enterocolitis in preterm neonates with very low birth weight: a systematic review of randomised controlled trials Lancet 2007 369 9573 1614 1620 10.1016/S0140-6736(07)60748-X 17499603

[B34] Johnston BC Goldenberg JZ Vandvik PO Sun X Guyatt GH Probiotics for the prevention of pediatric antibiotic-associated diarrhea Cochrane Database Sys Rev 2011 Art. No.: CD004827 Issue 11 10.1002/14651858.CD004827.pub3 22071814

[B35] Higgins JPT Green S Cochrane Handbook for Systematic Reviews of Interventions 2008 John Wiley & Sons, Chichester (UK)

[B36] Boehm G Supplementation of a bovine milk formula with an oligosaccharide mixture increases counts of faecal bifidobacteria in preterm infants Arch Dis Child Fetal Neonatal Ed 2002 86 F178 F181 10.1136/fn.86.3.F178 11978748 PMC1721408

[B37] Knol J Boehm G Lidestri M Negretti F Jelinek J Agosti M Stahl B Mosca F Increase of faecal bifidobacteria due to dietary oligosaccharides induces a reduction of clinically relevant pathogen germs in the feces of formula-fed preterm infants Act Paediatr 2005 Suppl 449 31 33 10.1111/j.1651-2227.2005.tb02152.x 16214763

[B38] Boehm G Fanaro S Jelinek J Stahl B Marini A Prebiotic concept for infant nutrition Acta Paediatr 2003 92 Suppl 441 64 67 10.1111/j.1651-2227.2003.tb00648.x 14599044

[B39] Costalos C Enteral feeding of premature infants with Saccharomyces Boulardii Early Hum Dev 2003 74 89 96 10.1016/S0378-3782(03)00090-2 14580749

[B40] Stratiki Z Costalos C Sevastiadou S Kastanidou O Skouroliakou M Giakoumatou A Petrohilou V The effect of a bifidobacter supplemented bovine milk on intestinal permeability of preterm infants Early Hum Dev 2007 83 575 579 10.1016/j.earlhumdev.2006.12.002 17229535

[B41] Reuman PD Duckworth DH Smith KL Kagan R Bucciarelli R Ayoub E Lack of effect of Lactobacillus on gastrointestinal bacterial colonization in premature infants Pediatr Infect Dis 1986 5 663 668 10.1097/00006454-198611000-00013 3099269

[B42] Indrio F Riezzo G Raimondi F Biscegua M Cavallo L Francailla R The effects of probiotics on feeding tolerance, bowel habits and gastrointestinal motility in preterm new-borns J Pediatr 2008 152 801 806 10.1016/j.jpeds.2007.11.005 18492520

[B43] Indrio F Riezzo Raimondi F Francavilla R Montagna O Valenzano M Cavallo L Boehm G Prebiotics improve gastric motility and gastric electrical activity in preterm new-borns J Pediatr Gastroenterol Nutr 2009 49 258 261 10.1097/MPG.0b013e3181926aec 19561548

[B44] Mihatsch WA Hoegel J Pohlandt F Prebiotic oligosaccharides reduce stool viscosity and accelerate gastrointestinal transport in preterm infants Acta Paediatr 2006 95 843 848 10.1080/08035250500486652 16801182

[B45] Kapiki A Costalos C Oikonomidou C Triantafyllidou A Loukatou E Pertrohilou The effect of a fructooligosaccharide supplemented formula on gut flora of preterm infants Early Hum Dev 2007 83 335 339 10.1016/j.earlhumdev.2006.07.003 16978805

[B46] Jacobs S The use of probiotics to reduce the incidence of sepsis in premature infants Australian New Zealand Clinical Trials Registry ACTRN126070001444415 26/02/2007 [www.anzctr.org.au]

[B47] Lozano JM Rojas M Prophylactic Probiotics in Premature infants Clinical trials registry, NCT00727363 2008 [www.clinicaltrials.gov]

[B48] Al-Hosni M Duenas M Ferrelli K Howard D Soll R Probiotics-supplemented feeding in extremely low birth weight infants Pediatric Academic Society Conference Proceedings at Vancouver Convention Centre, 2010 Abstract Number 1670.8: Course number 1670.(www.pas-meetings.org; www.abstract2view.com)

[B49] Patole S A randomized placebo controlled trial on the safety and efficacy of a probiotic product in reducing all case mortality and definite Necrotising Enterocolitis in preterm very low birth weight neonates Australian New Zealand Clinical Trials Registry, ACTRN12609000374268 27/05/2009 (www.anzctr.org.au)

[B50] Underwood M The impact of oligosaccharides and bifidobacteria on the intestinal micro flora of premature infants Clinical trials registry NCT00810160 05/11/2009. [www.clinicaltrials.gov]

[B51] Karvonen AV Sinkiewicz G Connoly E Vesikari T Safety and colonization of the probiotic Lactobacillus reuteri ATCC 55730 in new born and premature infants 2002 Bio Gaia AB Research Laboratories, Stockholm, Sweden (Unpublished data)

[B52] Agarwal R Sharma N Chaudhry R Deorari A Paul V Gewolb IH Panigrahi P Effects of oral Lactobacillus GG in enteric micro flora in low birth weight neonates J Pediatr Gastroenterol Nutr 2003 36 397 402 10.1097/00005176-200303000-00019 12604982

[B53] Lin HC Hsu CH Chen HL Chung MY Hsu JF Lien RI Tsao LY Chen CH Su BH Oral Probiotics prevent necrotizing enterocolitis in very low birth weight preterm infants: A multicenter, randomized controlled trial Pediatrics 2008 122 693 700 10.1542/peds.2007-3007 18829790

[B54] Riskin A Hochwald O Bader D Srugo I Naftali G Kugelman A Cohen E Mor F Kaufman B Shaoul R The effects of Lactulose supplemented enteral feedings in premature infants: A pilot study J Pediatr 2010 156 209 214 10.1016/j.jpeds.2009.09.006 19879595

[B55] Andrews BF Low birth weight infants fed a new carbohydrate- free formula with different sugars Am J Clin Nutr 1969 22 845 850 5797051 10.1093/ajcn/22.7.845

[B56] Chou IC Kuo HT Chang JS Wu SF Chiu HY Su BH Lin HC Lack of effects of oral probiotics on growth and neuro developmental outcomes in preterm very low birth weight infants J Pediatr 2010 156 393 396 10.1016/j.jpeds.2009.09.051 19914635

[B57] Stansbridge EM Walker V Hall MA Smith SL Millar MR Bacon C Chen S Effects of feeding premature infants with Lactobacillus GG on gut fermentation Arch Dis Child 1993 69 488 492 10.1136/adc.69.5_Spec_No.488 8285751 PMC1029590

[B58] Cukrowska B Lodinova-Zadnikiva R Enders C Sonnenborn U Schulze J Tlaskalová-Hogenová H Specific proliferative and antibody responses of premature infants to intestinal colonization with non-pathogenic probiotic E-coli strain nissle 1917 Scan J Immunol 2002 55 204 209 10.1046/j.1365-3083.2002.01005.x 11896937

[B59] Bin-Nun A Bromiker R Wilschanski M Kaplan M Rudensky B Caplan M Hammerman C Oral probiotics prevent necrotising enterocolitis in very low birth weight neonates J Pediatr 2005 147 192 196 10.1016/j.jpeds.2005.03.054 16126048

[B60] Manzoni P Mostert M Leonessa ML Priolo C Farina D Monetti C Latino MA Gomirato G Oral supplementation with Lactobacillus caseii subspecies rhamnosus prevents enteric colonization by candida species in preterm infant: A randomized study Clin Infec Dis 2006 42 1735 1742 10.1086/504324 16705580

[B61] Rouge C Piloquet H Butel MJ Berger B Rochat F Ferraris L Des RC Legrand A de la Cochetière MF N’Guyen JM Vodovar M Voyer M Darmaun D Rozé JC Oral Supplementation with probiotics in very low birth preterm infants: A randomized, double blind, placebo controlled trial Am J Clin Nutr 2009 89 1828 1835 10.3945/ajcn.2008.26919 19369375

[B62] Taylor SN Basile LA Ebeling M Wagner C Intestinal Permeability in Preterm infants by feeding type: Mother’s milk versus formula Breastfeed Med 2009 4 11 15 10.1089/bfm.2008.0114 19196035 PMC2932544

[B63] Hoyos AB Reduced Incidence of necrotizing enterocolitis associated with enteral administration of lactobacillus acidophilus and bifidobacterium infantis to neonates in an intensive care unit Int J Infect Dis 1999 3 197 202 10.1016/S1201-9712(99)90024-3 10575148

[B64] Wang C Shoji H Sato H Nagata S Ohtsuka Y Shimizu T Yamashiro Y Effects of Oral Administration of bifidobacterium breve on fecal lactic acid and short chain fatty acids in low birth weight infants J Pediatr Gastroenterol Nutr 2007 44 252 257 10.1097/01.mpg.0000252184.89922.5f 17255840

[B65] Dani C Biadaioli R Bertini G Martelli E Rubaltelli F Probiotics feeding in prevention of urinary tract infection, bacterial sepsis and necrotizing enterocolitis in preterm infants. A prospective double blind study Biol Neonate 2002 82 103 108 10.1159/000063096 12169832

[B66] Millar MR Bacon C Walker V Hall MA Enteral feeding of premature infants with Lactobacillus GG Arch Dis Child 1993 69 483 487 10.1136/adc.69.5_Spec_No.483 8285750 PMC1029589

[B67] Samanta M Sarkar M Ghosh P Ghosh JK Sinha MK Chatterjee S Prophylactic probiotics for prevention of necrotizing enterocolitis in very low birth weight new borns J Trop Pediatr 2009 55 128 131 18842610 10.1093/tropej/fmn091

[B68] Lidestri M Agosti M Marini M Oligosaccharides might stimulate calcium absorption in formula fed preterm infants Acta Paediatr 2003 92 Suppl 441 91 92 14599050 10.1111/j.1651-2227.2003.tb00654.x

[B69] Kitajima H Sumida Y Tanaka R Yuki T Fujimura M Early Administration of Bifidobacterium breve to preterm infants: Randomised controlled trial Arch Dis Child 1997 76 F101 F107 10.1136/fn.76.2.f101 PMC1720633 9135288

[B70] Mohan R Koebnick C Schildt J Schildt S Mueller M Possner M Radke M Blaut M Effects of Bifidobacterium lactis Bb12 supplementation on intestinal microbiota of preterm infants: A double blind, placebo controlled randomized study J Clin Microbio 2006 44 4025 4031 10.1128/JCM.00767-06 PMC1698302 16971641

[B71] Westerbeek EAM van Elburg RM Van den Berg A Van den Berg J Twisk JWR Fetter WPF Lafeber HN Design of a randomised controlled trial on immune effects of acidic and neutral oligosaccharides in the nutrition of preterm infants: Carrot study BMC Pediatr 2008 8 46 10.1186/1471-2431-8-46 18947426 PMC2579424

[B72] Lee SJ Cho SJ Park EA Effects of Probiotics on enteric flora and feeding tolerance in preterm infants Neonatology 2007 91 174 179 10.1159/000097449 17377402

[B73] Mohan R Koebnick C Schildt J Mueller M Radke M Blaut M Effects of Bifidobacterium lactis Bb12 supplementation on body weight, fecal pH, acetate, lactate, calprotectin and IgA in Preterm infants Pediatr Res 2008 64 418 422 10.1203/PDR.0b013e318181b7fa 18552710

[B74] Westerbeek EAM Van den Berg JP Lafeber HN Fetter-Wilem PF Boehm G Twisk WR Van Elburg RM Neutral and acidic oligosaccharides in preterm infants: a randomized double-blind, placebo controlled trial Am J Clin Nutr 2010 91 679 686 10.3945/ajcn.2009.28625 20032496

[B75] Lin HC Su BH Chen AC Lin TW Tsai CH Yeh TF Oh W Oral probiotics reduce the incidence and severity of necrotizing enterocolitis in very low birth weight infants Pediatrics 2005 115 1 4 15629973 10.1542/peds.2004-1463

[B76] Patole SK Muller R Does Carboxy methylcellulose have a role in reducing time to full enteral feeds? Int J Clin Pract 2005 59 544 548 15857350 10.1111/j.1742-1241.2004.00353.x

[B77] Yong G Fang H Shuang-Gen M Guo-Cheng X Effect of Bifid triple viable on feeding Intolerance in preterm infants with very low birth weight. [Clinical study on the effect of probiotic preparation on feeding intolerance in preterm infants with very low birth weight. (from Chinese translation)] Chinese Journal of Microecology 2009 21 451 452

[B78] van Elburg RM Fetter WPF Bunkers CM Heymans HSA Intestinal permeability in relation to birth weight and gestational and postnatal age Arch Dis Child Fetal Neonatal Ed 2003 88 F52 F55 10.1136/fn.88.1.F52 12496227 PMC1755997

[B79] Corpeleijn WE van Elburg RM van Kema IP Goudoever JB Assessment of intestinal permeability in (premature) neonates by sugar absorption tests Methods Mol Biol 2011 763 95 104 10.1007/978-1-61779-191-8_6 21874446

[B80] Westerbeek EAM van den Berg A Lafeber HN Fetter WPF van Elburg RM The effect of enteral supplementation of a prebiotic mixture of non-human milk galacto-, fructo- and acidic oligosaccharides on intestinal permeability in preterm infants Br J Nutr 2011 105 268 274 10.1017/S0007114510003405 20863418

[B81] Agostoni C Axelsson I Braegger C Goulet O Koletzko B Michaelsen KF Rigo J Shamir R Szajewska H Turck D Weaver L Probiotic Bacteria in dietetic products for infants: a commentary by the ESPGHAN Committee on Nutrition J Pediatr Gastroenterol Nutr 2004 38 365 374 10.1097/00005176-200404000-00001 15085012

[B82] Guenther K Straube E Pfister W Guenther A Huebler A Severe sepsis after probiotic treatment with Escherichia coli nissle 1917 Pediatr Infect Dis J 2010 29 188 189 20118747 10.1097/INF.0b013e3181c36eb9

[B83] Kunz AN Noel JM Fairchok MP Two cases of Lactobacillus bacteremia during probiotic treatment of short gut syndrome J Pediatr Gastroenterol Nutr 2004 38 457 458 10.1097/00005176-200404000-00017 15085028

[B84] Land MH Rouster-Stevens K Woods CR Cannon ML Cnota J Shetty AK Lactobacillus sepsis associated with probiotic therapy Pediatrics 2005 115 178 181 15629999 10.1542/peds.2004-2137

[B85] Veereman G Pediatric applications of inulin and oligofructose J Nutr 2007 137 2585S 2589S 17951508 10.1093/jn/137.11.2585S

